# The Use of Orthologous Sequences to Predict the Impact of Amino Acid Substitutions on Protein Function

**DOI:** 10.1371/journal.pgen.1000968

**Published:** 2010-05-27

**Authors:** Nicholas J. Marini, Paul D. Thomas, Jasper Rine

**Affiliations:** 1 California Institute for Quantitative Biosciences, Department of Molecular and Cellular Biology, University of California Berkeley, Berkeley, California, United States of America; 2 Evolutionary Systems Biology Group, SRI International, Menlo Park, California, United States of America; University of Michigan, United States of America

## Abstract

Computational predictions of the functional impact of genetic variation play a critical role in human genetics research. For nonsynonymous coding variants, most prediction algorithms make use of patterns of amino acid substitutions observed among homologous proteins at a given site. In particular, substitutions observed in orthologous proteins from other species are often assumed to be tolerated in the human protein as well. We examined this assumption by evaluating a panel of nonsynonymous mutants of a prototypical human enzyme, methylenetetrahydrofolate reductase (MTHFR), in a yeast cell-based functional assay. As expected, substitutions in human MTHFR at sites that are well-conserved across distant orthologs result in an impaired enzyme, while substitutions present in recently diverged sequences (including a 9-site mutant that “resurrects” the human-macaque ancestor) result in a functional enzyme. We also interrogated 30 sites with varying degrees of conservation by creating substitutions in the human enzyme that are accepted in at least one ortholog of MTHFR. Quite surprisingly, most of these substitutions were deleterious to the human enzyme. The results suggest that selective constraints vary between phylogenetic lineages such that inclusion of distant orthologs to infer selective pressures on the human enzyme may be misleading. We propose that homologous proteins are best used to reconstruct ancestral sequences and infer amino acid conservation among only direct lineal ancestors of a particular protein. We show that such an “ancestral site preservation” measure outperforms other prediction methods, not only in our selected set for MTHFR, but also in an exhaustive set of *E. coli* LacI mutants.

## Author Summary

The rapid pace of technological advances in DNA sequencing methods is leading to the discovery of genetic variants at a remarkable rate. Indeed, it is conceivable that entire individual genomes will be sequenced routinely in the near future. While these platforms greatly increase our ability to catalog variation, they are also creating a downstream need to efficiently process and filter this information to ultimately identify genetic causes underlying human disease. Since empirical evaluation of the biological effects of mutation is not practical at such a scale, computational methods that predict such effects are needed. In this paper, we describe a novel methodology to predict whether mutations that lead to amino acid substitutions in proteins will impact protein function and, therefore, may be more likely to have physiological consequences. Specifically, we use orthologous proteins to reconstruct the likely sequences of ancestral proteins in the human lineage. We found that the longer a position has been preserved from direct ancestors in the lineage leading to the human enzyme, the more likely that mutation at that site will have a deleterious effect. We demonstrated that the method should be generally applicable to all proteins.

## Introduction

Due to continuing advances in DNA sequencing technologies, our knowledge of human genetic variation is rapidly increasing. It is currently impractical to assay the biological effect of most genetic variants empirically, so computational predictions of their functional impact must play an important role in identifying potential genetic causes underlying human disease. Here, we focus on genetic variation that results in a single amino acid, or nonsynonymous, substitution in an encoded protein. Nonsynonymous changes comprise only a small fraction of known genetic variation, yet account for a disproportionately large fraction of known disease-causing variation in humans [1], [2].

It has long been recognized that amino acid substitutions that impair the function of a protein tend to involve substitution with a chemically very different amino acid [3], [4]. This is not surprising, as relatively few of the amino acids (e.g. active site amino acids of enzymes) in a protein are absolutely required for function. Most positions contribute to stabilizing the requisite structure of the active site and other binding and interaction sites, and such sites tend to be more tolerant of physico-chemically similar amino acid changes. As a result, some algorithms for predicting the functional effect of amino acid substitutions make use of measures of amino acid physico-chemical similarity. Multiple scales of physico-chemical similarity have been developed. The first, developed by Grantham [5], assigns to each possible amino acid substitution a similarity score that summarizes several physico-chemical properties of the two amino acid monomers. Grantham scores are site-independent (i.e. the substitution of a valine for an isoleucine is given the same score, regardless of the protein, or the site within the protein, in which the substitution occurs). However, it has been demonstrated that different sites vary greatly with respect to their tolerance to substitution. Miller and Kumar [4] demonstrated that human disease mutations have a strong statistical tendency to occur at evolutionarily conserved (i.e. slowly evolving) sites. Chasman and Adams [6] showed that mutation tolerance depends on several properties of the local 3D structure of the site, and this information has also been used to make site-specific predictions of the functional effects of substitutions [6], [7]. Such approaches are limited to proteins of known three-dimensional structure, or whose structure can be modeled based on a related protein.

The explosion in comparative genomics data has enabled site-specific predictions that are based on patterns of evolutionary substitution and that do not depend on structural information [8]–[12]. Given enough homologs, there is often considerable information about which substitutions have been “accepted” at a given site across the homologous proteins [13]. If a substitution was accepted (i.e. attained a high frequency in a population), the substitution had no appreciable negative impact on the fitness of the organism that harbored it, and therefore probably did not negatively impact protein function. The implicit assumption in deriving predictions based on this information is that accepted substitutions in one protein would also be accepted in its homologs; i.e. all homologs are assumed to be evolving under the same selective constraints. This assumption has recently been questioned for paralogous genes (genes that diverged by a gene duplication event presumably followed by some degree of functional divergence), and several groups have improved predictions by restricting analysis to orthologs, rather than paralogs [11], [14], [15]. However, it is not known to what extent orthologous genes share selective constraints, particularly after they have diverged substantially. In this paper, we test the utility of orthologs in the prediction of the functional effects of substitutions in the prototypical human enzyme methylenetetrahydrofolate reductase (MTHFR), which catalyzes a critical step necessary for the remethylation of homocysteine to methionine [16], [17].

We selected MTHFR as a model for testing these assumptions for several reasons. From a phenotypic standpoint, even mild defects in MTHFR can lead to metabolic imbalances that increase disease risk and thus would be subject to selective pressures. For example, a common polymorphism (677C→T) that changes an alanine at position 222 to a valine (referred to as A222V hereafter; [18]) impairs enzyme activity by 50% and leads to elevated plasma homocysteine levels in individuals with inadequate folate intakes [16]–[18]. Homocysteine may be a risk factor for several common diseases including cardiovascular disease [19] and neural tube defects [20]. From an evolutionary standpoint, MTHFR was present in the Last Universal Common Ancestor (LUCA) of all extant life, and has been recognizably conserved in nearly every organism sequenced to date. Since there appear to have been few gene duplications, the gene is present in single copy in nearly all animals, making these homologous genes unambiguous orthologs. Finally, a robust yeast complementation assay has been developed, allowing us to experimentally test the function of variants of MTHFR [21].

In addition, from MTHFR resequencing studies in random populations, we and others [21], [22] have identified many novel, low frequency nonsynonymous variants that affect enzyme function. Significantly, like the common A222V variant, many of these are remedial by folate supplementation [21]. As the pace of genetic discovery rapidly increases, there will surely be many novel MTHFR enzyme variants identified. Given the metabolic significance of MTHFR and the ability to nutritionally correct defective alleles, it would be desirable to predict *in silico* the functional impact of nonsynonymous mutation rather than empirically determine the effect of each variant individually. Furthermore, using MTHFR as a model enzyme may illustrate more general rules for computationally predicting the impact of amino acid substitution on protein function.

To this end, we constructed 35 MTHFR enzyme variants (34 of which differ from the major allele by a single amino acid and one represents a reconstruction of the human-macaque ancestral allele by a 9-site change) and tested them in a cell-based functional assay based on complementation in the yeast *Saccharomyces cerevisiae* [21]. Specifically, we targeted sites that have been conserved to varying degrees, and at these sites we created substitutions that have been accepted in at least one known ortholog of human MTHFR. We found, somewhat surprisingly, that most of our selected mutants of MTHFR were not tolerated in the human enzyme despite their presence in orthologous enzymes. These data suggested that the orthologous genes are evolving under different evolutionary constraints than human MTHFR. To remove potentially spurious signals from substitutions accepted in orthologs after their divergence from the human lineage, we subsequently restricted our analysis to only direct lineal ancestors of human MTHFR. Thus, we classified each site by its conservation only in direct ancestors of the human enzyme, which we refer to as “preservation” among ancestors to distinguish it from the common usage of “conservation” among homologs. The results indicated that the extent to which sites are preserved in lineal ancestors was a good predictor for whether that position was tolerable to change, not only for human MTHFR but also in the LacI protein from *E. coli*.

## Methods

### Quantitative Yeast Complementation Assay for MTHFR Function

**Figure 1:**
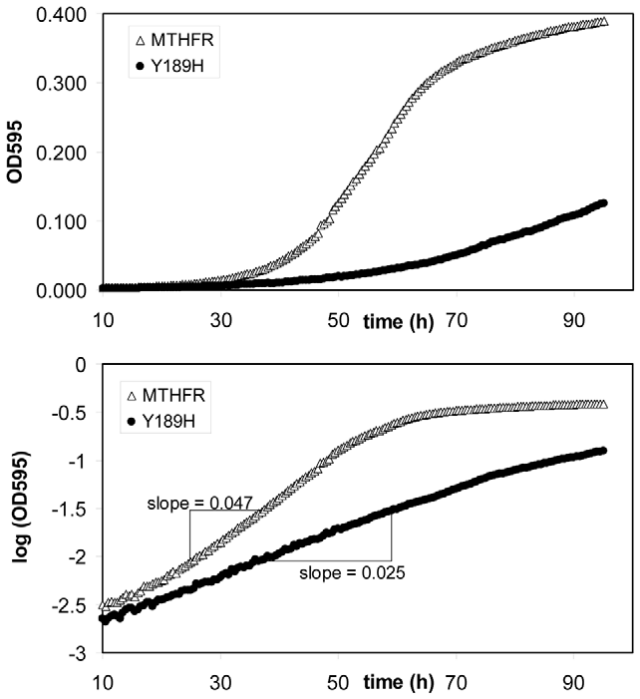
Example growth curves from which rate metrics were calculated. Shown are two examples (major MTHFR allele (open triangle); Y189H substitution variant (closed circle)) where growth in liquid culture was tracked over time, according to Methods. The upper panel shows absorbance (OD_595_) values and the lower panel shows the log_10_ transformation of the same absorbance reads. Log_10_-transformed data were used to calculate maximum slopes that served as growth-rate metrics.

The specific aspects of the assay have been described previously [21]. Briefly, a yeast strain was deleted for both the endogenous MTHFR (*MET13*, necessary for methionine synthesis) and for a folate biosynthetic enzyme (*FOL3*, dihydrofolate synthetase). The resulting strain (*met13::KanMX fol3::KanMX*) requires folate supplementation in the media and expression of a functional human MTHFR for growth in absence of methionine. Under the conditions of the assay, the rate at which the cells grow reflects the cellular activity of the MTHFR variant interrogated [21], [23] for any given level of folate supplementation. Following transformation of the parent strain by each individual variant, growth in the absence of methionine was recorded by optical density (OD_595_) measured over time in low-volume cultures supplemented with 25 ug/ml folinic acid [21]. To assign a growth-rate metric for quantitative comparisons between alleles, absorbance values were log_10_-transformed and a maximum slope was calculated which represented the maximal rate of cell growth (see Figure 1). Although this metric was not a cell doubling rate *per se*, slope calculations were easier to integrate into data handling, and relative differences between variants were exactly the same as they would be for doubling rates. All variants were tested in two or more experiments involving at least 5 replicates per experiment.

### Construction of Enzyme Variants

The yeast plasmid driving expression of human MTHFR variants under the inducible *GAL1* promoter has been described previously [21], [23]. This plasmid served as the backbone to reconstruct all MTHFR variants by site-directed mutagenesis using QuikChange kits (Stratagene). All variants were verified by sequencing the entire coding region of MTHFR.

### Defining Functional Versus Impaired Alleles

**Figure 2:**
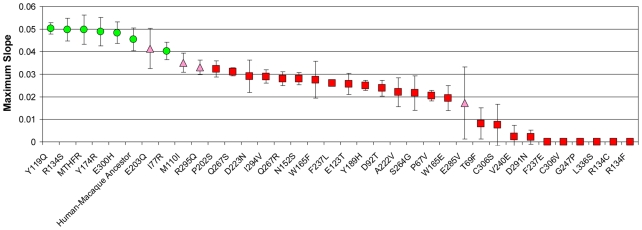
Activities of MTHR mutants. The average maximum slope (growth-rate metric) and standard deviation for each of the 36 MTHFR variants tested as in Methods. Replicate sets (N = 5) were compared against a positive control (major MTHFR allele) and a negative control (A222V allele) using 2 different statistical criteria as described in Methods. Green circles indicate changes not significantly different from the positive control and significantly better than the A222V control and indicate functionality. Red squares indicate changes significantly less active than the positive control and not significantly better than the A222V control and indicate impaired alleles. Pink triangles are classified as equivocal due to disagreement in the statistical methods. The raw replicate data and statistical metrics are in Table S1.

Replicate data sets for each substitution variant were compared against a positive control (major MTHFR allele) and a known impaired variant (A222V allele) as a negative control, using two different statistical criteria to evaluate whether variants were significantly different from either control. Significance was determined against each control using both a Student's t-test with a Bonferroni-corrected p-value (p<0.0014 for 35 pairwise comparisons) or Dunnett's test for comparing multiple treatments against a single control (alpha<0.01; [24]). Variants whose activity was not significantly different from the major allele and significantly better than the A222V allele *by both statistical measures* were classified as functional, whereas variants whose activity was significantly less than the major allele and not significantly better than the negative control were classified as impaired. In the cases where there was not a consensus in the comparisons, these alleles were classified as equivocal. In this way, of the 36 alleles tested in this study (including wild-type human MTHFR), 7 were classified as functional, 25 were classified as impaired and 4 were equivocal (see Figure 2). It should be noted that other statistical determinations of functionality are possible, but they do not appreciably change the results. For example, replacing Dunnett's test with False Discovery Rate analysis (q<0.01; [25]) results in the same classifications for 34 of the 36 alleles.

### Alignment of Orthologs, Phylogenetic Trees, and Ancestral Sequence Determination

#### Human MTHFR

The alignment and phylogenetic tree were from the PANTHER database [10], in which orthologs of human MTHFR were aligned using MAFFT [26], and a phylogenetic tree was constructed using the GIGA software (Thomas, submitted), which use s known species relationships to help infer the tree topology. Ancestral sequences were reconstructed for human MTHFR using the PAML software package (version 4.2; [27]) with the default parameter file for the aaml program (this uses the WAG empirical model to estimate amino acid substitution probabilities). For calculating ancestral site preservation (see below), we considered a site to have a “known” ancestral amino acid if the reconstruction probability exceeded 90%.

To more accurately reconstruct the human-macaque common ancestral sequence, we used the actual nucleotide coding sequences rather than the protein translation. We identified the predicted coding nucleotide sequences for MTHFR from the following Ensembl genomes: human, gorilla, orangutan, macaque, and mouse lemur. The sequences were aligned using MAFFT (with minor manual adjustment where necessary to maintain codon alignment), and the phylogenetic tree was assumed to match the species tree. Ancestral sequences were reconstructed using the codeml program from PAML with default parameters. This analysis does not use an empirical amino acid substitution model, but rather distinguishes between nucleotide transitions and transversions, and synonymous and nonsynonymous substitutions with a constant value of omega across the tree.

#### *E. coli* LacI

Orthologs of *E. coli* LacI were retrieved by a BLASTP search of the UniProt database, and choosing the top hits that sampled the bacterial clades useful for reconstructing ancestral residues. Included were several clades of enterobacteria, two clades of gamma-proteobacteria, and two actinobacteria as outgroups. A phylogenetic tree (Figure S1) was inferred using the Neighbor-Joining algorithm [28]. Ancestral sequences in the LacI family were determined using the aaml program within PAML as described above for the MTHFR family.

## Results

### Measuring Enzyme Function by Quantitative Complementation

We have previously shown that human MTHFR enzyme activity can be accurately measured in a simple yeast cell growth assay whereby expression of the human enzyme (or enzyme variant) is asked to complement the methionine biosynthetic defect of yeast MTHFR deletions (*met13*). Thus, by identifying enzyme variants that are incapable of fully restoring growth in media lacking methionine (relative to wild-type MTHFR), we have identified naturally occurring nonsynonymous polymorphisms that impair enzyme function [21]. To allow quantitative comparisons between variants in this assay, we derived a growth-rate metric by determining maximum slopes from log_10_ transformed growth curve data (Figure 1). In general, growth rates measured in this manner are directly reflective of the cellular activity of the variant assayed [21].

Intracellular folate levels can remedy defective alleles of MTHFR in humans and this behavior can be recapitulated in the yeast assay [21]. While this is attractive from a therapeutic perspective, this suppression may confound functional assessment of mutant alleles since high folate supplementation can mask enzymatic defects. To avoid this, cultures were supplemented with a level of folate that was mildly rate-limiting for growth (25 ug/ml folinic acid), which allows subtle changes in MTHFR activity to be reflected in the growth readout [21]. Under these conditions, the growth rate driven by the major human allele is approximately 91% of that when folate is not rate-limiting (data not shown). The activities of the complete collection of 36 variants were assayed in this way (Figure 2; raw replicate data for these experiments is in Table S1).

### Evolutionary Validation of Cell-Based Functional Determinations: Expected Behaviors of Highly Conserved Sites and a Reconstructed Recent Ancestor

As described in Methods, we used the unchanged major allele of MTHFR (wild type) as a positive control and the impaired A222V allele as a negative control to define growth-rate boundaries for distinguishing functional from impaired variants. Although the A222V variant is not a complete loss-of-function allele, it was chosen as a negative control to effectively set the threshold of activity that distinguishes functional from impaired variants. This change has been well documented to cause a 50% loss in intrinsic activity [16], [22] that, importantly, can lead to metabolic dysfunction and thus would be subject to selective pressures. Even so, this variant has reached a high frequency in the human population (∼30% global frequency; http://www.ncbi.nlm.nih.gov/SNP/snp_ref.cgi?rs=1801133). Thus, selective pressures against this allele must be minimal and this level of activity is likely to be near the borderline of impairment, unless there is some undetected heterozygote advantage.

To establish that the assay and this classification scheme can reliably distinguish functional from impaired variants of MTHFR, and that, at least in straightforward cases, analysis of orthologs accurately predicts whether a given variant will be functional or impaired, we first tested the major human MTHFR allele and a 9-site mutant that “resurrects” the human-macaque ancestral MTHFR (S9G, L53M, Y89F, R132C, I496V, V578I, R594Q, T639A A650E). As expected, we found both human MTHFR and the putative human-macaque ancestral MTHFR to complement the yeast *met13* defect with approximately the same, relatively high level of activity (Figure 2). On the other hand, the A222V substitution resulted in significantly reduced activity. We previously demonstrated this allele is defective in this assay [21] and demonstrate here that the quantitative growth defect (rate metric  = 0.022 for A222V vs. 0.0497 for the major MTHFR allele) was in good agreement with the reduced level of activity.

**Figure 3:**
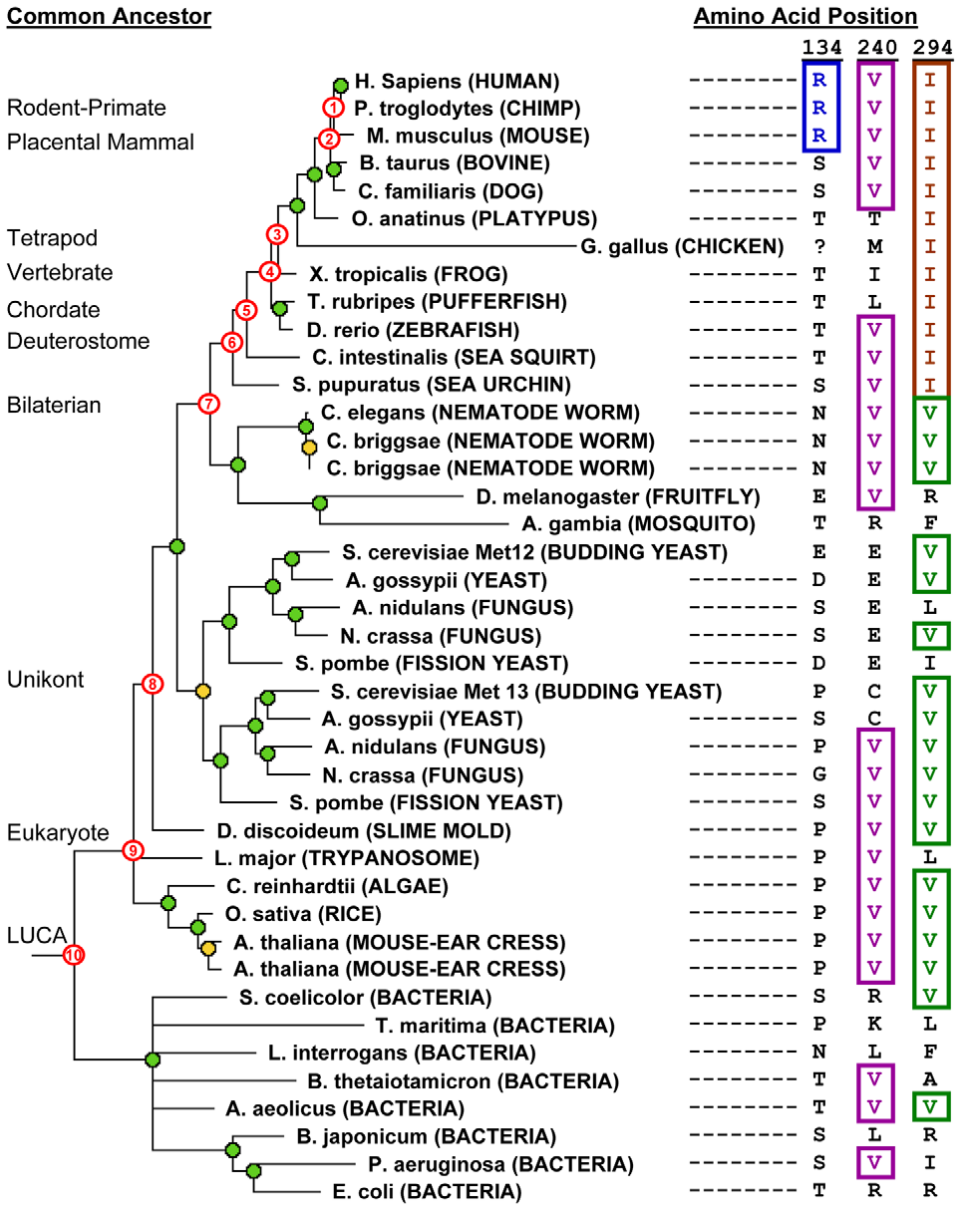
Phylogenetic tree and ancestral allele determination from orthologs of human MTHFR. Tree: MTHFR sequences from modern-day species are indicated. Database identifiers for these entries are listed in Table S2. Gene duplication events are shown with orange circles, and speciation events with green circles. Nodes numbered in red correspond to ancestral branch points in the human MTHFR lineage. Longer branch lengths indicate faster evolutionary rate. The chicken sequence was given an arbitrary, long branch length because it is a sequence fragment and the actual branch length could not be accurately determined. Ancestral allele determinations: The right columns show the amino acids found in the modern-day sequences corresponding to positions 134, 240 and 294 in human MTHFR. These are shown to illustrate how ancestral sites are determined and, consequently, how long the identity of the site in the human enzyme has been preserved in the human lineage (see text for details).

We subsequently constructed four single-site mutants of MTHFR that each alter a site that is highly conserved in nearly all orthologs and, thus, were expected to be functionally impaired (see Table S2 for sequence alignments at all sites interrogated in this study). For example, P67V substitutes a chemically very dissimilar amino acid at a site that is conserved among all known eukaryotic (and some prokaryotic) orthologs of human MTHFR and, as expected, was classified as impaired (rate metric  = 0.02). D291N targets an aspartic acid residue present in nearly all eukaryotes and despite the chemical similarity of aspartate and asparagine, the D291N variant was severely impaired (rate metric  = 0.002). Lastly, we tested two different substitutions of R134, a site at which no single amino acid is highly conserved, but at which only polar amino acids are observed across nearly all MTHFR orthologs (see Figure 3). R134C and R134F each substitute a hydrophobic amino acid and each result in a drastically impaired enzyme (both rate metrics  = 0). This is consistent with the prediction that negative selection against hydrophobic amino acids had occurred at this site, even though the identity is not strictly conserved. These examples confirm that our assay is capable of distinguishing between functional and impaired variants, and that, at least in very straightforward cases, evolutionary conservation among orthologs can also make this distinction.

### Testing Single-Site Substitutions that Are Accepted in Orthologs of Human MTHFR

We then selected 30 additional site-specific mutants to interrogate, based on an alignment of orthologs of human MTHFR. Each of the 30 sites was conserved among primates, but diverged to varying degrees in more distant lineages/species. Furthermore, at these sites we introduced substitutions that were accepted in at least one known ortholog (Table 1). We reasoned that these would be relatively difficult cases for prediction methods since this set represents sites that are both partially conserved and partially divergent and little is known how to balance these signals.

**Table 1:** Amino acid substitutions from human MTHFR orthologs tested for functional impact.

Change*	Human Site Conserved in Clade	Substitution Accepted in Ortholog**	Human Lineage Preservation***	Grantham score	SIFT score
Y119Q	Deuterostomes, Nematodes, ***N. crassa***	***A. nidulans*** *, A. niger* (Met12 group)	Bilaterian	99	0.21
R134S	***P. troglodytes, M. musculus***	***B. taurus, C. familiaris***	Rodent-Primate	110	0.66
Y174R	Deuterostomes, Fungi, ***D. discoideum***, Bacteria	***C. elegans, C. briggsae***	Unikont	77	0.12
E300H	Amniotes, Nematodes, Fungi	***S. purpuratus*** *, * ***A. thaliana***	Tetrapod	40	0.24
E203Q	Vertebrates, Fungi, Bacteria	***C. elegans, C. briggsae***	Vertebrate	29	0.11
I77R	Chordates	***N. crassa***, *B. fuckeliana* (Met12 group)	Vertebrate	97	0.1
M110I	Deuterostomes, ***L. major***	***S. pombe*** (Met12 group)	Deuterostome	10	0.19
R295Q	Deuterostomes, Fungi	***C. elegans, C. briggsae***	Deuterostome	54	0.06
P202S	Deuterostomes, Nematodes, Fungi, Plants, Bacteria	***D. discoideum, S. pombe*** (Met13 group)	Last Universal	74	0.08
Q267S	Chordates	***S. purpuratus***	Chordate/Last Universal#	80	0.02
D223N	All Clades	***C. elegans, C. briggsae***	Last Universal	23	0.13
I294V	Deuterostomes	***C. elegans, C. briggsae***	Deuterostome/Last Universal#	29	1
Q267R	Chordates	***C. elegans, C. briggsae***	Chordate/Last Universal#	54	1
N152S	Deuterostomes, Fungi, Bacteria	***C. elegans, C. briggsae***	Last Universal	46	0.04
W165F	Deuterostomes, Fungi, Bacteria	***A. thaliana, O. sativa***	Unikont	40	0.3
F237L	Placental Mammals	***G. gallus, X. tropicalis***	Mammalian/Eukaryote#	22	1
E123T	Vertebrates, Fungi, Plants, ***D.discoideum***	***A. nidulans, N. crassa*** (Met12 group)	Vertebrate	65	0.16
Y189H	Vertebrates, Nematodes, Fungi, Plants	***S. cerevisiae, A. nidulans, A. gossypii, N. crassa*** (Met13 group)	Eukaryote	83	0.56
D92T	Deuterostomes, Nematodes, Fungi, Plants	***S. cerevisiae, A. gossypii, A. nidulans*** (Met12 group)	Eukaryote	85	0.08
A222V	All Clades	***T. maritima*** *, T. petrophila,*	Last Universal	64	0.06
S264G	Bilaterians, Fungi	***D. discoideum, A. thaliana, O. sativa***	Bilaterian	56	0.41
W165E	Deuterostomes, Fungi, Bacteria	***A. gossypii*** *, P. pastoris* (Met12 group)	Unikont	152	0.11
E285V	All Clades	***S. pombe*** (Met13 group)	Last universal	121	0.02
T69F	Deuterostomes, Fungi, Plants, Bacteria	***C. elegans, C. briggsae***	Last Universal	103	0.04
C306S	Deuterostomes, Nematodes, Fungi, Plants, Bacteria	***D. discoideum*** *, T. pseudonana*	Last Universal	112	0.02
V240E	Deuterostomes, Nematodes, Fungi, Plants, Bacteria	***S. cerevisiae, A.gossypii, A.nidulans, S. pombe, N. crassa*** (Met12 group)	Last Universal	121	0.08
F237E	Placental mammals	***C. elegans, C. briggsae***	Mammalian/Eukaryote#	140	0.18
C306V	Deuterostomes, Nematodes, Fungi, Plants, Bacteria	***A. gossypii, A. nidulans*** (Met12 group)	Last Universal	192	0.09
G247P	All Clades	***N. crassa*** *, B. fuckeliana* (Met12 group)	Last Universal	42	0.03
L336S	All Clades	***S. cerevisiae, S. pombe, A. gossypii*** (Met12 group)	Last Universal	145	0.03

*Human-Macaque ancestral reconstruction and substitutions not found in any ortholog (P67V, R134C, R134F, D291N) are not listed.

**Boldface species are present in Figure 3.

***Ancestral reconstruction, >90% probability using PAML.

#The identities of these residues are conserved in the human lineage from the more recent of the two ancestors listed, at which point a longer-standing pattern of preservation (dating back to the more ancient of the two) was broken.

“Human Site Conserved in Clade” lists clades in which the identity of the human enzyme is seen multiple times. The sequence alignments for all positions interrogated in this study are in Table S2. “Human Lineage Preservation” is the most ancient MTHFR ancestor in which the identity of that site in the human enzyme has been preserved. “Substitution accepted in Ortholog”: MTHFR ortholog(s) in which the substitution can be found. Grantham score [5] estimates the physico-chemical dis-similarity between the parent and substituting amino acids. SIFT score [32] estimates whether the amino acid change can be tolerated based on the variability at that site in homologous proteins (see text for details). The order is presented as in Figure 2.

From a practical standpoint, we limited our changes to the catalytic domain of MTHFR, which comprises the N-terminal half of the 656 amino acid protein (NCBI protein reference NP_005958). This region is more prone to deleterious changes [21], [22] and most mutations that result in severe clinical phenotypes occur in the catalytic domain (http://www.hgmd.cf.ac.uk). In addition, although all genes shown in the phylogenetic tree (Figure 3) are orthologous to human MTHFR by the definition of Fitch [29], we did not choose amino acids accepted in insect orthologs. The insect lineage appears to have an accelerated evolutionary rate and has lost the C-terminal regulatory domain found in other eukaryotes, thus their functional orthology is questionable. Furthermore, we distinguished between the orthologs resulting from the gene duplication event in fungi as belonging to either the Met13 group (defined by *S. cerevisiae* Met13) or the Met12 group (defined by *S. cerevisiae* Met12). Evidence from *S. cerevisiae* and *S. pombe* suggests that the Met13 group of enzymes may be functionally more similar to human MTHFR than those in the Met12 group, which have diverged to a slightly greater degree [30], [31].

According to our functional definition, 21 mutants in this set of 30 had impaired activity, 5 were functional, and 4 were equivocal (Figure 2). The relatively large number of impaired mutants was unexpected given the results of computational prediction methods. For example, the SIFT (Sorting Intolerant From Tolerant) algorithm [32] predicted that only 7 mutants would be impaired using the recommended 0.05 score threshold (T69F, N152S, G247P, Q267S, E285V, C306S, L336S; Table 1). SIFT calculates a score for each mutant based on the substitutions observed at a given site across a set of homologous proteins. Like most existing methods that incorporate homology as a predictive parameter, SIFT assumes that orthologs are evolving under similar evolutionary constraints. Since all of our substitutions appear in an ortholog of human MTHFR, it is not surprising that SIFT's predictions are strongly biased toward functional substitutions. Concordant with this view, a second commonly used predictive algorithm, Pmut [12], also predicted 80% of these substitutions to be functional (data not shown). Since most changes were accepted in more than one closely related ortholog (Table 1), it is unlikely that the data is skewed by infrequent alleles or spurious mutation in the individual MTHFR genes sequenced.

**Figure 4:**
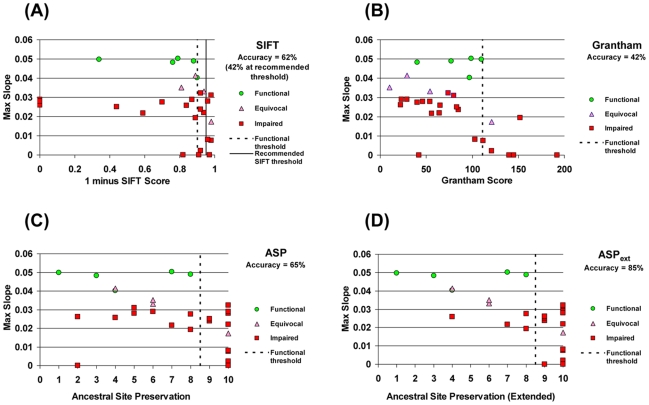
Accuracy of discrimination between functional and impaired variants by different methods. Growth-rate metrics for the 30 variants in Table 1 plotted against scores/classifications from various methods that estimate functional impact. The accuracy of each method was determined by calculating the number of mutations correctly called as functional or impaired divided by the number of mutations unambiguously classified by experimental data. Binning of mutations was determined by using a threshold empirically defined by the functional alleles (dashed vertical line in each panel) to define the functional (left of line) and impaired (right of line) bins. (A) SIFT score; note that the graph plots (1–score) to facilitate comparison with the other methods. All functional variants have a SIFT score >0.09 which, when used as a threshold results in a classification accuracy of 62%. The recommended threshold of 0.05 (solid vertical line) results in a lower classification accuracy (42%). (B) Grantham scale of amino acid dissimilarity between wild-type and substituted amino acid. (C) Ancestral Site Preservation (ASP) measure, using inferred ancestral sequences of human MTHFR. Numbers on the x-axis correspond to increasingly ancient ancestors of human MTHFR as defined by the nodes in Figure 3. (D) Ancestral Site Preservation Extended (ASP*_ext_*) measure. If a site was preserved in the ancestral lineage for a long period *before* being substituted by the current-day amino acid, the more ancient ancestor is used to define preservation at this site. The preservation measure for 5 variants is shifted by this criterion (see Table 1).

SIFT displayed only 42% accuracy in discriminating functional from impaired MTHFR alleles when considering only those changes unambiguously classified as functional or impaired in the yeast assay (i.e. ignoring the 4 equivocal calls in this 30 mutation set; Figure 4A). Since all functional alleles had a SIFT score > = 0.09, a threshold empirically set at this point (rather than the 0.05 recommended) would have increased overall accuracy to 62%. However, even this threshold would have still resulted in significant over-prediction of functional alleles (Figure 4A).

We next assessed a completely different distance metric than evolutionary conservation. The Grantham score provides a measure of the physico-chemical dis-similarity between any pair of amino acids [5]. This measure was evaluated against the observed growth rates for each of these 30 mutations (Figure 4B). Consistent with previous results [4], there was a significant negative correlation (R = −0.48, P_val_ = 0.007), indicating that, on average, the larger the physico-chemical difference between the wild-type and substituting amino acid, the greater the functional impairment. However, despite this correlation, the Grantham differences were not very useful for discriminating functional from impaired mutations: even a threshold optimized for our dataset yields an accuracy of only 42% (Figure 4B). Similarly to SIFT, such a stratification of the data would have led to an over-prediction of functional alleles. Thus, neither of these tools, which analyze and estimate the impact of amino acid substitution in very different ways, appeared particularly accurate on this dataset.

### Distinguishing Functional from Impaired Mutants in MTHFR: the Ancestral Site Preservation (ASP) Measure

Our finding that most of the mutants we tested were impaired, despite the presence of these substitutions in an orthologous protein, suggests that in these cases the site is not under the same selective constraint in human MTHFR as it is in the ortholog. In other words, selective constraints on a given site may differ between lineages in the phylogenetic tree. Therefore it may be more relevant to infer selective constraints by considering only the *direct ancestral lineage* of a given protein, rather than all lineages (as in methodologies such as SIFT that consider homologous sequences from other organisms). We therefore defined a measure (Ancestral Site Preservation or ASP) that is determined by the most distant ancestor in which a given site is preserved. To determine this, we first constructed a phylogenetic tree for the family and inferred ancestral sequences at specific nodes in the tree (Figure 3). We then traced back among the direct ancestors of the current-day sequences to infer how long a given amino acid had been preserved at a particular site. The ASP score is simply the (relative) number of the most ancient ancestor in which the identity of the site in human MTHFR is still preserved.

To illustrate this ASP score, Figure 3 provides examples of allele determinations in human MTHFR ancestors at three instructive positions in the human enzyme. Site 134 aligns an arginine (R) in human, chimp and mouse MTHFR and, thus, is inferred to be present in the rodent-primate ancestor (node #1 on tree). However, because no other orthologs align R at this position, we cannot infer the ancestral preservation prior to node #1, and the ASP score  = 1. The valine (V) at position 240, on the other hand, is extremely well-preserved in ancestors of human MTHFR. V is inferred to be present in the placental mammal ancestor (node #2 on tree) since all descendants align a V at that position. Furthermore, from analyzing increasingly distant outgroups from this node, the presence of a V in vertebrates (zebrafish), deuterostomes (sea urchin, sea squirt), worms and insects strongly suggests the presence of a V back to the bilaterian ancestor (node #7). Analysis of even more distant outgroups shows that V is also aligned at this position in several fungal orthologs, plants, *Leishmania*, *Dictyostelium* and several distant bacterial species. The most parsimonious inference, therefore, is that V240 is preserved in all ancestors of human MTHFR back to the LUCA (node #10), giving an ASP score of 10. The third example, position 294, reveals an interesting preservation pattern whereby preservation of isoleucine (I) from a relatively recent ancestor (inferred from the I present in all deuterostome species) broke a longer-standing pattern of ancestral preservation (V is inferred to be present in all more ancient human ancestors: bilaterian through LUCA; nodes 7–10).

For our set of mutants, we found that if the identity of the site was strictly preserved from at least as far back as the Most Recent Common Ancestor (MRCA) of all eukaryotic MTHFRs (node #9, Figure 3), a mutation at that site impaired human MTHFR. On the other hand, all benign changes that resulted in a functional enzyme were at sites whose identity was preserved from no further than the MRCA of all unikont MTHFRs (node #8 in Figure 3). Thus, the unikont MTHFR ancestor empirically defined the functional threshold for the ASP measure (Figure 4C). The ASP measure showed an accuracy of 65% among all unambiguously classified changes and performed as well or better than Grantham scores or SIFT for predicting functional and impaired variants in this dataset.

Furthermore, we observed that five impaired mutants that were mispredicted by ASP (F237L, F237E, Q267R, Q267S, I294V) occurred at 3 positions with a very particular pattern of ancestral preservation in which one amino acid was strictly preserved beginning with the eukaryotic MRCA or LUCA, and substituted only once after that. This is a very interesting pattern in light of our results and one that is not common across MTHFR sites in general. As mentioned above, isoleucine (I) 294 in human MTHFR is an instructive example (Figure 3). Valine is strictly preserved at this site in the human lineage beginning with the LUCA, though it does diverge in some other lineages (e.g. *L. major*). This strongly suggests the site was under selective constraint in the ancient ancestors of human MTHFR. However, this site was fixed as isoleucine beginning with the MRCA of all deuterostome MTHFRs, suggesting that this ancestral constraint had changed in the deuterostome MTHFR lineage, possibly due to positive selection. Indeed, a reversion to the ancestral amino acid, I294V in human MTHFR, resulted in a significant impairment of function (Figure 2). Likewise at position 267, glutamine (Q) in the human enzyme is preserved since the chordate common ancestor, which broke the long-standing preservation of arginine (R) at this site since the LUCA (Table 1; see Table S2 for alignment). At position 237, phenylalanine (F) is preserved since the placental mammal ancestor, which broke the long-standing preservation of leucine (L) from the eukaryote MRCA. As for I294V, changes reverting to the more ancient amino acid (Q267R and F237L) impaired human MTHFR, even though L237 is accepted in the recently diverged chicken ortholog (Table 1, Figure 2).

The pattern whereby a long-standing ancestral preservation is broken only once by a more recent ancestor in the human lineage indicates that such positions have been under selective constraint for long periods of time and that these constraints changed during evolution of the lineage. If so, these sites are likely to be more sensitive to substitutions even though the site in the human enzyme has been preserved from only a relatively recent ancestor. Thus, to capture the importance of such sites, we further define the Ancestral Site Preservation *Extended* measure (ASP*_ext_*). The ASP*_ext_* is similar to the ASP but traces back to an earlier ancestor if there was a previous long-standing (eukaryotic MRCA or earlier) strict preservation at that site. ASP*_ext_* significantly improves the ability to predict functional impairment among our set of MTHFR mutants (85% accuracy; Figure 4D).

### Generalizing ASP and ASP*_ext_* to Other Proteins: Predicting Functional and Impaired Mutants of LacI

To determine if the ASP would be a useful measure for predicting functional and impaired variants more generally, we tested its predictive ability against the extensive *E. coli* lac repressor (LacI) substitution dataset [33], [34]. LacI is a standard test-case for prediction algorithms because it contains nearly comprehensive substitution data: the functional impact of 12–13 nonsynonymous mutants at each of 328 sites in the LacI protein was determined by *in vivo* assay. Furthermore, this data set, comprising a total of 4041 single-site mutations, was used as a training set for the SIFT algorithm [35], so SIFT has been optimized to perform on these data. We reasoned that if ASP could perform well on LacI predictions, despite not being optimized for this specific data set, this would be good evidence of its more general utility. We would not necessarily expect ASP to perform as well as SIFT, simply because ASP makes relatively crude predictions: a site is predicted to be either completely tolerant (all mutations are predicted to be functional) or completely intolerant (all mutations are predicted to be impaired). On the other hand, SIFT can predict different outcomes for different amino acids at each site.

We were somewhat surprised, then, to find that both ASP and ASP*_ext_* actually out-performed SIFT on predictions of LacI mutants (Table 2). In this analysis, we constructed a phylogenetic tree for LacI in bacterial species and inferred the ancestral identities of amino acids at divergence points in the tree, as we did for MTHFR (Figure S1). As described above, we empirically defined the common ancestor to unikonts as the “threshold ancestor” for stratifying functional and impaired alleles of MTHFR: all benign changes were at sites preserved for this length of time or less. Interestingly, the prokaryotic threshold ancestor that yielded the highest prediction accuracy was the common ancestor of LacI proteins in *E. coli* and *Enterobacter cancerogenus*. LacI proteins in these two species share 52% identity, which is approximately the same overall divergence as between human MTHFR and its *D. discoideum* ortholog (48%) which both share the unikont ancestor (Figure 3). This suggests a simple method for setting a general threshold across different domains of life.

**Table 2:** Prediction accuracy of different algorithms on the LacI dataset.

Algorithm	Accuracy	Reference*
SIFT (v.1)	68.3%	35
SIFT (v.2)	68.1%	9
MAPP (including paralogs)	69.2%	11
MAPP (only orthologs)	70.7%	11
ASP	72.0%	This study
ASP extended	72.0%	This study

*Only studies with published prediction results on essentially all of the >4000 mutations in the LacI dataset are listed.

As shown in Table 2, the accuracy of both ASP and ASP*_ext_* was 72% when each substitution in LacI is simply classified as functional (wild-type levels) or impaired (less than wild-type levels), as is commonly done [9], [11], [35]. There was no difference in performance between ASP and ASP*_ext_* as only 4 sites differed between the two methods. The 72% accuracy rate exceeds the best prediction accuracy, to our knowledge, previously reported on the LacI test set (70.7%), obtained by the MAPP method (Multivariate Analysis of Protein Polymorphism; 11) trained on LacI and five of its orthologs. Interestingly, MAPP reportedly performed worse (69.2%) when trained using the full set of 55 LacI homologs, which included numerous putative paralogs. Thus, restricting the training set to a few orthologs minimizes the opportunity for divergence of selective constraints in different lineages, which is consistent with our findings for MTHFR.

## Discussion

Most algorithms for the functional prediction of amino acid substitution assume that divergent lineages share the same selective constraints on homologous sites. However, we observed that replacing a single amino acid in the human MTHFR enzyme with one that was accepted in an orthologous protein frequently resulted in functional impairment. Indeed, we observed that even a substitution from a very recently diverged ortholog (F237L, which is fixed in chicken MTHFR) resulted in significant functional impairment. These results suggested that selective constraints can vary in divergent lineages and do not necessarily reflect those directing evolution of the human enzyme.

Our results are in agreement with previous studies that suggest that the selective constraints on homologous sites may differ substantially among orthologous proteins. There are several reasons that selective constraints may differ between lineages, including changes in the strength (from differences in population dynamics that arise from the inability of selection to remove weakly deleterious mutations from a smaller population) or even the direction of selection (e.g. from differences in gene essentiality for different organisms; [36]). However, several studies have concluded that the most likely reason is compensatory mutations, i.e. the change in selective constraints on a given site is actually due to mutations at other sites in the same (or possibly another) protein. Kondrashov et al. [37] estimated that about 10% of all amino acid substitutions producing a pathogenic phenotype in humans are present as the wild-type amino acid in at least one mammalian ortholog. The authors termed these types of substitutions “compensated pathogenic deviations” and suggested that they were tolerated in the orthologous protein because of second-site compensatory mutations. In support of this hypothesis, Gao and Zhang [38] provided evidence that compensatory mutations are the most likely explanation for the majority of human disease mutations fixed in mice. Kulathinal et al. [39] found that debilitating missense mutations in *D. melanogaster* were fixed in related insect orthologs at a surprisingly high rate, and also speculated that compensatory mutations must be co-evolving in these lineages. Of the substitutions we tested, we found that 70% were functionally impaired, a much greater proportion than reported in the related studies cited above. However, it should be noted that the majority of the substitutions we tested were from more distant orthologs (26 of 30 substitutions were fixed in orthologs whose MRCA with human was over 500 million years ago). The long divergence times have provided more opportunity for compensatory mutations to arise that alter the selective constraints on a given site.

The term “compensatory mutation” may appear to suggest that chronologically a second mutation occurred that “compensates” for a pre-existing deleterious mutation. In fact, the compensatory mutation most likely arises first as a “pre-adaptation” that enables a mutation that would otherwise have been deleterious. Thus, our findings are consistent with the importance of evolutionary trajectory in determining which substitutions are permissable at any given point during evolution. For example, using ancestral protein reconstruction, Zhang and Rosenberg [40] identified two amino acid substitutions that contributed to the evolutionary enhancement of RNase activity of EDN, an eosinophil-associated RNase of primates. Each substitution individually was neutral or perhaps only mildly deleterious to ancestral function, but in combination were complementary in the evolution of a derived, modified function. Likewise, neutral substitutions at some sites in the mineralocorticoid receptor were found to be critical for enabling adaptive substitutions to occur at other sites [41]. In addition, multiple amino acid substitutions in beta lactamase that confer antibiotic resistance seem to arise through only a very small fraction of available trajectories [42]. In other words, the identity of an amino acid at one site can be influenced by the content of other sites and these relationships may be unique to the evolution of specific lineages. These findings highlight the importance of previous substitutions in defining the possibilities of subsequent mutational events.

Given these considerations, we hypothesized that selective constraints on a site might be better estimated from only the evolutionary trajectory of a particular protein (or gene), rather than including substitutions among divergent lineages. This would remove potentially confounding signals from these other lineages, such as differences in population dynamics, adaptation to specific environments and compensatory mutations. To test our hypothesis, we constructed a phylogenetic tree, and inferred ancestral sequences at specific nodes in the tree for the MTHFR family as well as the bacterial LacI family. We could then trace back among the direct ancestors of the current-day sequence, to infer how long a given amino acid had been preserved at a particular site, which we dubbed the Ancestral Site Preservation (ASP) measure. In support of our hypothesis, we found that sites that are preserved for long periods of time in the human lineage (high ASP) will not tolerate substitution, even though such substitutions are tolerated in at least one orthologous protein. In addition, the ASP metric discriminated between functional and impaired variants in both human MTHFR and *E. coli* LacI (using a comparable threshold in both cases) more accurately than any method reported to date. This was surprising given that the ASP does not consider the nature of the specific amino acid substitution, but rather only the ancestry of a particular site in the protein. This suggests that, to a reasonable approximation, most sites within a protein are either intolerant or tolerant with respect to a wide variety of mutations, and that intolerant and tolerant sites can be discriminated quite well using the evolutionary history of substitution at each site. It is also perhaps somewhat surprising that the ancestral preservation must be for a very long time before it can be considered to be reliably indicative of negative selection against variant amino acids. In MTHFR, all substitutions we tested at sites preserved since at least the eukaryotic common ancestor (approximately 1500 million years ago), undoubtedly due to selection against variant amino acids, resulted in a nonfunctional protein by our definition. However, when preservation has occurred for shorter periods of time, most mutations we tested had no significant functional impact in our assay, suggesting that in MTHFR ancestral preservation even for many hundreds of millions of years may be due in part to random chance rather than selection.

For MTHFR we found that a modified measure, which we dubbed “ASP *extended*” (ASP*_ext_*), improved the quality of predictions by accounting for sites that had been preserved for long periods of time during the history of MTHFR, but then changed relatively recently. These are examples of changed evolutionary constraints *within* the human lineage and, as discussed above for extant orthologous proteins, are most likely due to compensatory mutations. ASP*_ext_* is capable of highlighting substitutions fixed by recent positive selection in the human lineage (which has been suggested; [36], [43]), if they occurred at previously preserved sites.

Thus, we conclude that as orthologs diverge from their most recent common ancestor, their different evolutionary trajectories lead to the divergence in the selective constraints on homologous sites. While lineage-specific adaptive selection and variable strength of purifying selection contribute to such divergence of constraints, the accumulation of potentially compensatory neutral (or near-neutral) substitutions underlie and enable lineage-specific trajectories. In support of this conclusion, Kondrashov et al. [44] have recently shown that the continued sequence divergence observed for distant orthologs is inconsistent with a model of lineage-independent selective constraints on homologous sites.

In this study we defined preservation strictly in that only the identity of the amino acid at a particular site was considered in determining the ASP. Of course, this definition could be relaxed to include chemically similar amino acids. As we discussed above, there seemed to be a strict requirement for a polar amino acid at position 134 in human MTHFR, although the identity of this site (arginine) is preserved back to only the rodent-primate ancestor. Thus, using polarity as the preservation feature rather than identity would yield a much larger ASP score and may be more informative for changes such as R134F and R134C which are not polar and are quite debilitating. In this case, the R134S change, which was functional, would still be predicted to be functional because it does not break the polarity preservation.

These observations have implications for understanding and reconstructing protein evolution, as well as improving the accuracy of predicting the effects of amino acids substitution on human protein function. The ASP measure appeared to have advantages over other comparative sequence approaches in using phylogeny to predict functionality, though it remains clear that physico-chemical constraints on substitution also play a role. Use of our basic approach should prevent distinct historical constraints from confounding inferences about physico-chemical constraint, and reveal how to better incorporate both ancestral preservation and physico-chemical differences into functional prediction. Perhaps most importantly, by enabling more accurate computational predictions of functional polymorphisms in humans, our results should aid epidemiological efforts in identifying the genetic deficiencies that are etiological for disease.

## Supporting Information

Figure S1 Phylogenetic tree of bacterial orthologs of the Lac repressor (LacI). UniProt identifiers and species are listed. ARTS2 = *Arthrobacter*, strain FB24 (actinobacteria); STRCO = *Streptomyces coelicolor* (actinobacteria); PSEU2 = *Pseudomonas syringae* (gamma-proteobacteria); HAEIN = *Haemophilus influenzae* (gamma-proteobacteria); YERPE = *Yersinia pestis* (enterobacteria); 9ENTR = *Enterobacter cancerogenus* (enterobacteria); CITRO = *Citrobacter* sp. 30_2 (enterobacteria); SALAR *Salmonella arizonae* (enterobacteria); ECOLI = *Escherichia coli* K12 (enterobacteria); ECO7I = *Escherichia coli* O7:K1 (enterobacteria). Arrow indicates the common ancestor between *E. coli* and *Enterobacter* and was used as the ancestral threshold for predicting functional or impaired alleles.
(0.04 MB DOC)

Table S1 Maximum slopes for individual replicates and statistical calculations.
(0.07 MB DOC)

Table S2 Multiple sequence alignments.
(0.04 MB XLS)
