# Supplementary material for: The Use of Orthologous Sequences to Predict the Impact of Amino Acid Substitutions on Protein Function
Source: PLoS Genet. 2010 May 27;6(5):e1000968. doi: 10.1371/journal.pgen.1000968 (PMC2877731; doi:10.1371/journal.pgen.1000968)
Supplement: Figure S1 — Phylogenetic tree of bacterial orthologs of the Lac repressor (LacI). UniProt identifiers and species are listed. ARTS2 = Arthrobacter, strain FB24 (actinobacteria); STRCO = Streptomyces coelicolor (actinobacteria); PSEU2 = Pseudomonas syringae (gamma-proteobacteria); HAEIN = Haemophilus influenzae (gamma-proteobacteria); YERPE = Yersinia pestis (enterobacteria); 9ENTR = Enterobacter cancerogenus (enterobacteria); CITRO = Citrobacter sp. 30_2 (enterobacteria); SALAR Salmonella arizonae (enterobacteria); ECOLI = Escherichia coli K12 (enterobacteria); ECO7I = Escherichia coli O7:K1 (enterobacteria). Arrow indicates the common ancestor between E. coli and Enterobacter and was used as the ancestral threshold for predicting functional or impaired alleles. (0.04 MB DOC) [file pgen.1000968.s001.doc]

**Figure S1. Phylogenetic tree of bacterial orthologs of the Lac repressor (LacI).** UniProt identifiers and species are listed. ARTS2=*Arthrobacter*, strain FB24 (actinobacteria); STRCO=*Streptomyces coelicolor* (actinobacteria); PSEU2=*Pseudomonas syringae* (gamma-proteobacteria); HAEIN=*Haemophilus influenzae* (gamma-proteobacteria); YERPE=*Yersinia pestis* (enterobacteria); 9ENTR=*Enterobacter cancerogenus* (enterobacteria); CITRO=*Citrobacter* sp. 30_2 (enterobacteria); SALAR *Salmonella arizonae* (enterobacteria); ECOLI=*Escherichia coli* K12 (enterobacteria); ECO7I=*Escherichia coli* O7:K1 (enterobacteria). Arrow indicates the common ancestor between *E. coli* and *Enterobacter* and was used as the ancestral threshold for predicting functional or impaired alleles.


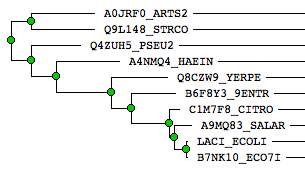


Threshold Ancestor
