# Supplementary material for: The Use of Orthologous Sequences to Predict the Impact of Amino Acid Substitutions on Protein Function
Source: PLoS Genet. 2010 May 27;6(5):e1000968. doi: 10.1371/journal.pgen.1000968 (PMC2877731; doi:10.1371/journal.pgen.1000968)
Supplement: Table S1 — Maximum slopes for individual replicates and statistical calculations. (0.07 MB DOC) [file pgen.1000968.s002.doc]

**Table S1. Maximum Slopes for Individual Replicates and Statistical Calculations.**

| Change | Replicate1 | Replicate2 | Replicate3 | Replicate4 | Replicate5 | Average | Standard Deviation | P-value VS Major Allele | Dunnett's q VS Major Allele | P-value VS A222V | Dunnett's q VS A222V | Functional Designation |
| --- | --- | --- | --- | --- | --- | --- | --- | --- | --- | --- | --- | --- |
| Y119Q | 0.050174 | 0.049591 | 0.048714 | 0.054733 | 0.048485 | 0.050339 | 0.002548 | 0.83605 | -0.1937 | 0.00001 | 8.2521 | Functional |
| R134S | 0.056544 | 0.053464 | 0.047333 | 0.044140 | 0.047381 | 0.049772 | 0.005071 | 0.97942 | -0.0285 | 0.00006 | 8.0869 | Functional |
| MTHFR (Major Allele) | 0.058592 | 0.053215 | 0.047380 | 0.041555 | 0.047631 | 0.049675 | 0.006470 | 1.00000 | 0.0000 | 0.00013 | 8.0584 | Functional |
| Y174R | 0.051645 | 0.051223 | 0.047053 | 0.055535 | 0.038833 | 0.048858 | 0.006358 | 0.84545 | 0.2380 | 0.00015 | 7.8204 | Functional |
| E300H | 0.056544 | 0.046341 | 0.047333 | 0.044140 | 0.047381 | 0.048348 | 0.004766 | 0.72160 | 0.3865 | 0.00007 | 7.6719 | Functional |
| Human-Macaque Ancestor | 0.049750 | 0.041263 | 0.050264 | 0.039168 | 0.047144 | 0.045518 | 0.005038 | 0.28979 | 1.2112 | 0.00019 | 6.8472 | Functional |
| E203Q | 0.032929 | 0.032924 | 0.051520 | 0.040189 | 0.049474 | 0.041407 | 0.008841 | 0.13000 | 2.4089 | 0.00401 | 5.6495 | Equivocal |
| I77R | 0.045472 | 0.041375 | 0.037236 | 0.041862 | 0.035607 | 0.040310 | 0.003929 | 0.02444 | 2.7285 | 0.00058 | 5.3299 | Functional |
| M110I | 0.035851 | 0.030985 | 0.038986 | 0.030366 | 0.039304 | 0.035099 | 0.004263 | 0.00297 | 4.2471 | 0.00498 | 3.8113 | Equivocal |
| R295Q | 0.035964 | 0.036906 | 0.031763 | 0.029183 | 0.031774 | 0.033118 | 0.003224 | 0.00091 | 4.8241 | 0.00812 | 3.2343 | Equivocal |
| P202S | 0.034851 | 0.028008 | 0.028985 | 0.034486 | 0.035304 | 0.032327 | 0.003526 | 0.00076 | 5.0546 | 0.01292 | 3.0038 | Impaired |
| Q267S | 0.032415 | 0.028527 | 0.032562 | 0.029689 | 0.032188 | 0.031076 | 0.001848 | 0.00026 | 5.4190 | 0.01524 | 2.6394 | Impaired |
| D223N | 0.017681 | 0.038933 | 0.033206 | 0.023300 | 0.032038 | 0.029031 | 0.007172 | 0.00139 | 6.0148 | 0.13586 | 2.0436 | Impaired |
| I294V | 0.033150 | 0.030333 | 0.026857 | 0.028762 | 0.025590 | 0.028938 | 0.002969 | 0.00019 | 6.0420 | 0.05762 | 2.0164 | Impaired |
| Q267R | 0.024961 | 0.024380 | 0.031025 | 0.029786 | 0.030014 | 0.028033 | 0.003112 | 0.00015 | 6.3058 | 0.09277 | 1.7526 | Impaired |
| N152S | 0.023535 | 0.028107 | 0.030137 | 0.028030 | 0.030244 | 0.028010 | 0.002718 | 0.00012 | 6.3124 | 0.08743 | 1.7460 | Impaired |
| W165F | 0.021271 | 0.017889 | 0.036498 | 0.027446 | 0.034403 | 0.027502 | 0.008175 | 0.00136 | 6.4606 | 0.26949 | 1.5978 | Impaired |
| F237L | 0.026227 | 0.028405 | 0.025435 | 0.024518 | 0.026071 | 0.026131 | 0.001438 | 0.00005 | 6.8599 | 0.19388 | 1.1985 | Impaired |
| E123T | 0.027460 | 0.032078 | 0.019960 | 0.022702 | 0.026553 | 0.025751 | 0.004648 | 0.00015 | 6.9708 | 0.31861 | 1.0876 | Impaired |
| Y189H | 0.026480 | 0.021330 | 0.025030 | 0.025028 | 0.027426 | 0.025059 | 0.002320 | 0.00004 | 7.1724 | 0.34231 | 0.8860 | Impaired |
| D92T | 0.026479 | 0.019689 | 0.022241 | 0.022386 | 0.028372 | 0.023833 | 0.003515 | 0.00005 | 7.5295 | 0.59015 | 0.5289 | Impaired |
| A222V | 0.024499 | 0.021707 | 0.029715 | 0.012300 | 0.021869 | 0.022018 | 0.006324 | 0.00013 | 8.0584 | 1.00000 | 0.0000 | Impaired |
| S264G | 0.033318 | 0.013282 | 0.020508 | 0.023596 | 0.017549 | 0.021651 | 0.007551 | 0.00023 | 8.1654 | 0.93558 | -0.1070 | Impaired |
| P67V | 0.016867 | 0.022121 | 0.022807 | 0.020776 | 0.019680 | 0.020450 | 0.002339 | 0.00001 | 8.5152 | 0.61715 | -0.4569 | Impaired |
| W165E | 0.017304 | 0.021922 | 0.013253 | 0.017015 | 0.027613 | 0.019421 | 0.005515 | 0.00005 | 8.8150 | 0.50855 | -0.7566 | Impaired |
| E285V | 0 | 0 | 0.030842 | 0.030754 | 0.024890 | 0.017297 | 0.015973 | 0.00299 | 9.4339 | 0.55598 | -1.3755 | Equivocal |
| T69F | 0 | 0.004058 | 0.016298 | 0.014168 | 0.006064 | 0.008118 | 0.006894 | 0.00001 | 12.1086 | 0.01050 | -4.0502 | Impaired |
| C306S | 0 | 0 | 0.011559 | 0.004520 | 0.021442 | 0.007504 | 0.009114 | 0.00003 | 12.2873 | 0.01912 | -4.2289 | Impaired |
| V240E | 0.011245 | 0 | 0 | 0 | 0 | 0.002249 | 0.005029 | 0.00000 | 13.8185 | 0.00059 | -5.7601 | Impaired |
| D291N | 0 | 0 | 0.003681 | 0.006990 | 0 | 0.002134 | 0.003148 | 0.00000 | 13.8520 | 0.00023 | -5.7936 | Impaired |
| F237E | 0 | 0 | 0 | 0 | 0 | 0 | 0 | 0.00000 | 14.4738 | 0.00005 | -6.4155 | Impaired |
| C306V | 0 | 0 | 0 | 0 | 0 | 0 | 0 | 0.00000 | 14.4738 | 0.00005 | -6.4155 | Impaired |
| G247P | 0 | 0 | 0 | 0 | 0 | 0 | 0 | 0.00000 | 14.4738 | 0.00005 | -6.4155 | Impaired |
| L336S | 0 | 0 | 0 | 0 | 0 | 0 | 0 | 0.00000 | 14.4738 | 0.00005 | -6.4155 | Impaired |
| R134C | 0 | 0 | 0 | 0 | 0 | 0 | 0 | 0.00000 | 14.4738 | 0.00005 | -6.4155 | Impaired |
| R134F | 0 | 0 | 0 | 0 | 0 | 0 | 0 | 0.00000 | 14.4738 | 0.00005 | -6.4155 | Impaired |
